# Machine-assessed tar yield marketing on cigarette packages from two cities in South Korea

**DOI:** 10.18332/tid/136421

**Published:** 2021-06-25

**Authors:** Michael Iacobelli, Juhee Cho, Kevin Welding, Kate Smith, Joanna E. Cohen

**Affiliations:** 1 Institute for Global Tobacco Control, Department of Health, Behavior and Society, Johns Hopkins Bloomberg School of Public Health, Baltimore, United States; 2 Department of Clinical Research Design and Evaluation, The Samsung Advanced Institute for Health Sciences and Technology, Sungkyunkwan University, Seoul, South Korea; 3 Department of Health, Behavior and Society, Johns Hopkins Bloomberg School of Public Health, Baltimore, United States

**Keywords:** packaging, branding, South Korea, misleading descriptors

## Abstract

**INTRODUCTION:**

South Koreans continue to smoke at high rates. Tobacco manufacturers have a history of branding cigarettes with misleading descriptors including the introduction of low or ultra-low tar brand variants. The government bans traditional misleading descriptors (low, mild) but requires the presence of machine-assessed tar yields on cigarette packages. Literature suggests the presence of quantitative constituents can be misleading for smokers. We analyzed the machine-assessed tar value branding and the presence of additional branding that highlight tar levels on South Korean cigarette packs.

**METHODS:**

In August 2018, we analyzed 178 unique cigarette packs purchased in Seoul and Busan, South Korea using a systematic protocol. Cigarette packs were coded for tar levels and classified as ultra-low, low, mid, and high tar. The presence of misleading descriptors and any additional branding relating to tar or potentially indicating strength were also coded.

**RESULTS:**

Machine-assessed tar yields ranged from 0.1 to 8 mg. Cigarettes with a 1 mg machine-assessed tar yield accounted for 38% of all packs purchased. A majority (80%) of packs with tar values <3 mg had additional marketing present on the pack that highlighted tar yields, compared to 45% for packs with tar values 3 mg or greater. Many (85%) of the 1 mg packs and all of the 0.1 and 0.5 mg packs had additional marketing present that referenced tar levels.

**CONCLUSIONS:**

These findings suggest that tobacco manufacturers are highlighting and reinforcing the tar yields of lower tar cigarettes by the deliberate use of tar level branding. These actions have the potential to mislead South Korean consumers that some cigarettes are less harmful than others. Strengthening of tobacco packaging regulations to prohibit references to tar yields on packs are needed to further protect consumers.

## INTRODUCTION

South Korea’s smoking rate remains high; in 2017, 37% of men and 5.2% of women smoked cigarettes^1^. South Korea requires a 50% graphic health warning label covering the front and back principal display areas, and since 1988 has banned the use of misleading descriptors (e.g. low, mild)^2^. The complete list of terms banned on cigarette packaging is left to the discretion of the President, which are ‘light’, ‘mild’, ‘low tar’, and ‘mild/gentle’. Additionally, any symbol, figures, drawings, and three-dimensional shapes – or a combination of these– that are similar to the aforementioned terms that attempt to minimize the health hazards caused by cigarettes, and as a consequence may induce incorrect perceptions about cigarettes are prohibited^2^. However, the government also requires that tobacco manufacturers list the tar and nicotine of cigarettes, as measured by ‘a measurement agency designated by the Minister of Strategy and Finance’ on the side of the package^3^. This requirement is inconsistent with the Framework Convention on Tobacco Control (FCTC) Article 11 guidelines which assert that ‘Parties should not require quantitative’ statements about tobacco constituents and emissions as these might imply that one brand is less harmful than another^4^.

South Korea currently does not have a comprehensive smoke-free policy, with only healthcare and educational facilities being protected^5^. While cigarette packs are required to carry a 50% graphic warning label, many countries have implemented larger warning labels and/or introduced plain and standardized packaging. The Korean government currently has a cigarette excise tax of 64.76% of the retail price of a pack, below the 70% minimum outlined by the WHO^1^. The continued high rates of smoking in South Korea should prompt policymakers to employ as many of the evidence-based recommendations of the FCTC as possible, including comprehensive smoke-free policies as well as stricter packaging regulations, including the prohibition of as many permutations of misleading descriptors found on cigarette packaging as possible, including quantitative statements of tobacco constituents, or plain and standard packaging.

Tobacco companies promote products using various potentially misleading strategies. For instance, studies have demonstrated that consumers misperceive that some cigarettes are less risky than others based on the machine-assessed product yields of harmful and potentially harmful constituents printed on tobacco product packaging and labeling^6-9^. Industry documents have shown that smokers who are having difficulty quitting might switch to a ‘low tar’ brand rather than quit^10^. In one study conducted with South Korean smokers, removing the machine-assessed tar yields from cigarette packaging reduced smokers’ misconceptions about ‘low tar’ cigarettes^11^. However, even with prohibitions on the display of machine-assessed cigarette constituents, cigarette brands are known to utilize color-coding and/or other design elements to communicate machine-assessed tar yields^12^.

The Korean tobacco industry is dominated by four companies: KT&G, Philip Morris Korea (PMK), Japanese Tobacco International, Korea (JTI), and British American Tobacco (BAT). KT&G accounts for just over 60% of all retail cigarette volume in South Korea^13^.

The tobacco industry has a long history of marketing low tar cigarettes and implicitly promoting these products as healthier alternatives^14^. ‘Low tar’ can connote different machine-assessed tar yields in different countries. For example, in China, 13 mg is considered ‘low tar’^15^, whereas in Japan, cigarettes with machine-assessed tar yields ranging from 1 to <6 mg are considered ‘ultra-lights’^16^. In South Korea, KT&G denote ‘ultra-low’ tar as cigarettes with machine-assessed tar yields of <1 mg^17^ and ‘low tar’ are cigarettes with machine-assessed yields of ≥1 mg but <3 mg. ‘Low tar’ cigarettes account for over 90% of all cigarette sales in South Korea since at least 2010^13^. The top three selling brand variants by retail volume in South Korea in 2018 were three Esse products, one 0.1 mg and two 1 mg cigarettes^13^.

The practice of marketing cigarettes with the machine-assessed tar yield is common in South Korea, with retailers utilizing tar yields to differentiate among different cigarette brand variants on sales receipts^18^. The presence of branding elements that reinforce the machine-assessed tar yield to imply that cigarettes are ‘low tar’ or ‘safer’, such as the inclusion of tar yields in the brand name are also deceptive^7^. We conducted a content analysis of cigarette packs purchased in South Korea to determine the extent of branding corresponding to the required machine-assessed tar yields printed on the side of the package.

## METHODS

In August 2018, we adapted a systematic protocol for collecting and analyzing cigarette packs^19^ to attempt to purchase as many unique cigarette packs available for sale at the time of data collection. We purchased cigarette packs from the two largest cities in South Korea: Seoul and Busan. Within each city we stratified neighborhoods by middle and high socioeconomic status (SES) and collected from nine neighborhoods (4 middle and 5 high SES) in Seoul and 4 neighborhoods in Busan (1 middle and 3 high SES). The SES of each neighborhood sampled was confirmed using residential real estate prices (price/m^2^) obtained from Real Estate 119, a domestic real estate listing website^20^. Neighborhoods were also selected for geographical diversity.

Data collection in each neighborhood started at a pre-identified hub. A systematic walking protocol from the hub was used to identify vendors that represented the top venue types where people purchase their cigarettes in South Korea (determined by Euromonitor). The walking protocol included alternating which vendor type purchases were made to increase the chances of finding unique packs in each neighborhood. A cigarette pack was considered unique if there was at least one difference in an exterior feature of the pack including: stick count, size, brand name presentation, brand variant name, colors, cellophane, packaging material (i.e. hard, soft, thin), and inclusion of a promotional item. Every unique pack encountered was purchased.

The neighborhood SES composition is a deviation to the published protocol in that no low-income neighborhoods were sampled. The primary intention of cigarette purchases was to identify product innovations that the tobacco industry is introducing in South Korea. To that end, we decided to purchase tobacco products from only middle- and high-income neighborhoods where young adults live and frequently visit, such as where universities are located and in popular nightlife districts, to most efficiently identify possible innovations released by tobacco companies. The examination of branding related to the machine-assessed tar value reported on the package emerged as an important finding of this pack collection.

### Coding of misleading descriptors on cigarette packages

Cigarette packs were coded for the presence of machine-assessed tar yields. Cigarette packs were also coded for the presence of traditional misleading descriptors (e.g. low, mild), any adjacent misleading descriptors (e.g. soft, smooth, mellow), and for the presence of any additional branding references to the machine-assessed tar yield (referred to as ‘tar number branding’ throughout) on the package, including any numbers or symbols potentially indicating strength. Any further information that may reinforce the additional tar number branding (such as the presence of qualitative descriptions of tar levels or references to milligrams or mg) to the machine-assessed tar yield was also captured.

Cigarettes were classified as ‘ultra-low’ tar if they had a listed machine-assessed tar yield <1mg; low tar for 1 to <3 mg; mid tar for 3 to < 6 mg; and high tar for ≥6 mg. This classification was based on the ranges used by KT&G^15^. Cigarette packs were double coded by trained coders. Any discrepancies were resolved by a third coder. A chi-squared test of independence was performed using STATA 14.2 (College Station, TX, USA) to examine the relationship between the presence of tar level branding and the machine-assessed tar yield of the cigarette.

In addition to coding the cigarette packs, the top brand family from each of the major tobacco companies (Esse, KT&G; Marlboro, PMK; Dunhill, BAT; Mevius, JTI) were qualitatively assessed to examine color, logo, design, or any other features that might suggest a lower harm product.

## RESULTS

### Machine-assessed tar yields

In total, 182 unique cigarette packs were collected. The analytic sample consisted of 178 unique cigarette packs that had the current government mandated graphic warning label, 4 packs (2%) featured an old Korean warning label and were excluded; all cigarette packs had a machine-assessed yield for tar on the side of the package. None of the cigarettes purchased had any lexical references to traditional misleading descriptors, such as ‘mild’ or ‘low tar’. However, 17% (n=31) packs had lexical terms that were adjacent to traditional misleading terms, such as soft, smooth, and mellow. These adjacent misleading terms were present among all tar level groups (ultra-low, n=1; low, n=14; mid, n=11; high, n=5). A chi-squared test of independence among all four groups was not significant [χ^2^(3)=0.6385, p=0.888].

**Figure 1:**
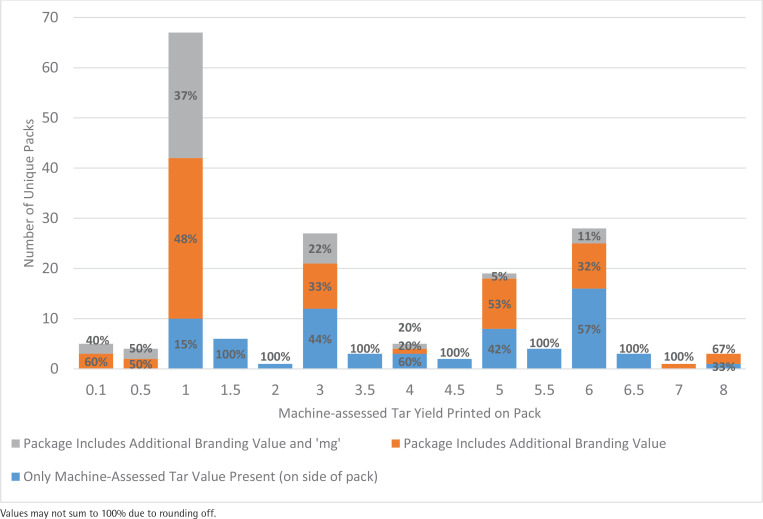
Presence of machine-assessed tar number branding on unique cigarette packs purchased in Seoul and Busan, South Korea, 2018

Among all cigarette packs purchased, 5% (n=9) had machine-assessed tar yields that were classified as ultra-low tar (<1 mg); 42% (n=74) had machine-assessed tar yields that were classified as low tar (1 to <3 mg); 34% (n=60) had machine-assessed tar yields that were classified as mid tar (3 to <6 mg); and 20% (n=35) had machine-assessed tar yields that were classified as high tar (≥6 mg). No cigarette packs had machine-assessed tar values greater than 8 mg (Figure 1). Only KT&G and BAT offered cigarettes in the ultra-low tar range (Supplementary file Table S1).

Ultra-low tar and low tar cigarettes combined accounted for almost half (47%) of all cigarette packs in the sample. Cigarette packs with a 1 mg machine-assessed tar yield represented the largest proportion (38%, n=67) of all pack presentations in the sample. Cigarette packs with machine-assessed tar yields of 6 mg (16%), 3 mg (15%), and 5 mg (11%) represented the next three most prevalent tar yields among the unique packs in our sample.

A chi-squared test of independence was performed to examine the relationship between the presence of tar level branding and the machine-assessed tar yield of the cigarette. The relationship across all four tar level groups was significant [χ^2^(3)=23.8165, p<0.001]. The relationships were also significant between the ultra-low and mid groups [χ^2^(1)=8.9514, p=0.003], ultra-low and high [χ^2^(1)=9.4286, p=0.002], low and mid [χ^2^(1)=13.1670, p<0.001], low and high [χ^2^(1)=12.3731, p<0.001]. The relationships between ultra-low and low [χ^2^(1)=2.6001, p=0.107] and mid and high [χ^2^(1)=0.1295, p=0.719] were not significant.

### Presence of additional branding on packaging

**Figure 2:**
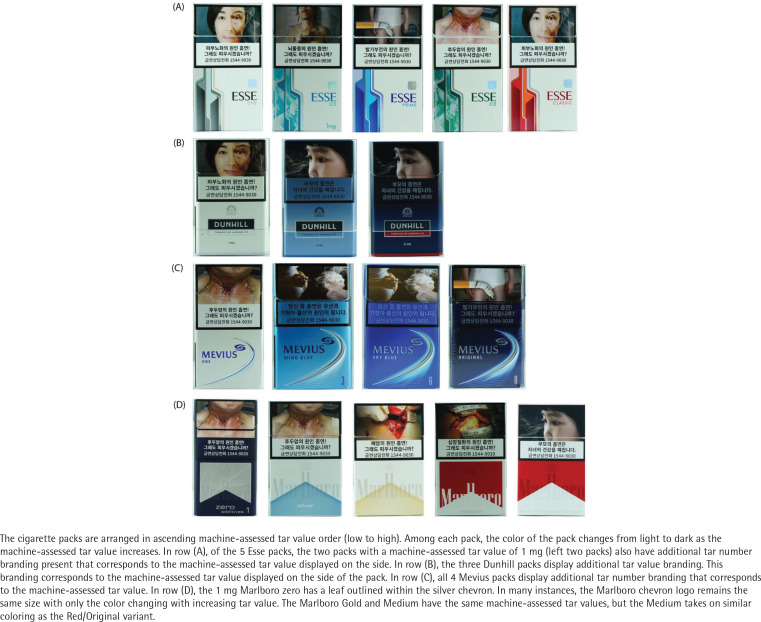
Images of cigarette packs purchased in Seoul and Busan, South Korea, 2018

One hundred and nine (61%) packs displayed tar number branding that matched the machine-assessed tar yield printed on the side of the package (Table 1). Forty of the 109 cigarette packs (37%) also had a milligrams (mg) symbol present alongside this tar number. No other symbols (including dots that indicate strength) were observed on the packages. No packs were observed to have a milligrams symbol in the absence of tar number branding. The milligrams symbol was most likely to be present on the front of the cigarette packages (80%, n=32) (Table 1, Figure 2).

**Table 1:** Location frequency of additional tar number branding and the presence of milligrams unit ‘mg’ on unique cigarette packs purchased in South Korea

	*Tar value group*	*Location of additional tar number branding and presence of ‘mg’*					
		*Front n (%)*	*Back n (%)*	*Top n (%)*	*Bottom n (%)*	*Sides n (%)*	*Bevel n (%)*
**Additional tar number branding**	Ultra-low (n=9)	9 (100)	4 (44)	5 (56)	5 (56)	-	-
	Low (n=57)	51 (89)	32 (56)	25 (44)	23 (40)	12 (21)	1 (2)
	Mid (n=28)	25 (89)	18 (64)	15 (54)	15 (54)	8 (29)	1 (4)
	High (n=15)	13 (87)	9 (60)	4 (27)	5 (33)	4 (27)	1 (7)
	Total (n=109)	98 (90)	63 (58)	49 (45)	48 (44)	24 (22)	3 (3)
**Presence of ‘mg’**	Ultra-low (n=4)	4 (100)	1 (25)	3 (75)	3 (75)	-	-
	Low (n=25)	20 (80)	13 (52)	9 (36)	5 (20)	2 (8)	-
	Mid (n=8)	6 (75)	3 (38)	3 (38)	2 (25)	-	1 (13)
	High (n=3)	2 (67)	-	-	-	-	1 (33)
	Total (n=40)	32 (80)	17 (43)	15 (38)	10 (25)	2 (5)	2 (5)

Observations are not mutually exclusive; most cigarette packs had multiple instances of additional branding present on the package.

Every ultra-low tar cigarette in our sample (100%, n=9) had tar number branding that matched the machine-assessed tar yield printed on front of the pack, with the branding also appearing on the top (56%), bottom (56%) and back (44%) of the pack. Almost half (44%, n=4) of ultra-low tar cigarettes purchased also had ‘mg’ included beside the tar number branding. For all tar levels, the front of the pack was the prime location for additional tar number branding appearing on 90% (n=98) of all cigarette packs (Figure 1).

Many of the cigarettes (85%, n=57) with a machine-assessed tar yield of 1 mg had tar number branding of ‘one’ or ‘1’ somewhere on the package. Twenty-five (37%) 1 mg cigarettes also had a ‘mg’ displayed alongside the additional tar number branding. Cigarettes with a machine-assessed tar yield of 3 mg (56%, n=15/27), 5 mg (58%, n=11/19), and 6 mg (43%, n=12/28) also had high proportions of packs that displayed an additional tar number branding. Every 0.1 mg (n=5), 0.5 mg (n=4), and 7 mg (n=1) pack purchased had an additional tar number branding present (Supplementary file Table S2).

### Qualitative assessment of top brand families

The four brand family variants explored were Esse (KT&G), Marlboro (PMK), Dunhill (BAT), and Mevius (JTI). Three packs were purchased within the Esse ‘bamboo’ subfamily. Within this subfamily, the color of the pack is white, but the color of the bamboo on the front of the pack gets increasingly darker: from light blue (0.1 mg reported tar yield), to green-yellow (0.5 mg reported tar yield), to darker green (1 mg reported tar yield). There are no variant names that distinguish this line, simply the color of the bamboo. Within the main Esse brand line, the main color of the package was always white, but the branding along the left front of the package changed color with increasing tar yield: One, a 1mg tar yield with light gray; Prime, a 4.5 mg tar yield pack with blue; and Classic, a 6.5 mg tar yield pack with red (Figure 2).

Similar color shifts were evident among the Dunhill packs (1 mg reported tar yield pack being white, the 3 mg reported tar yield pack displaying a light blue and the 6 mg tar yield pack displaying a dark blue color), but no variant names were present.

Among the Mevius packs, the main color of the package shifted from white to a dark blue. The variant names were One (white 1 mg tar), Wind Blue (light blue 3 mg tar), Sky Blue (darker blue 6 mg tar), and Original (darkest blue 8 mg tar).

Within the Marlboro brand family, a ‘zero additive’ variant anchored the lowest tar yield with 1mg in a black pack with a leaf outline within the chevron. The 3 mg ‘silver’ variant had light blue branding, the 6 mg ‘gold’ variant had gold branding, and the 8 mg variant had red with no variant name present on the pack. There was one additional 6 mg Marlboro variant called ‘medium’ which had the same color red branding as the 8 mg variant, but the chevron was smaller.

## DISCUSSION

Each of the major tobacco companies in South Korea employ similar branding tactics using color to communicate to users the relative ‘strength’ of the product. These tactics have been elucidated elsewhere^6-9,12^. What these data show is that in South Korea, tobacco companies are complying with the requirement of displaying machine-assessed constituent values as well as leveraging those values to communicate the relative ‘strength’ of their products using more overt branding. While cigarettes we purchased were found to have branding elements that included references to the machine-assessed tar yield at every level, cigarettes labeled as 1 mg tar accounted for a majority of the instances where such practices occurred, suggesting a conscious effort to market these products to new or existing consumers.

The reporting of machine-assessed cigarette constituents, such as tar and nicotine, have an impact on how smokers perceive the brands they are consuming^6,7^. Even with wide spread prohibition of traditional misleading descriptors such as ‘low’ and ‘mild’, the use of machine-assessed tar yields on packaging can promote switching to a lower tar brand variant as an alternative to quitting^9,10^. Previous experimental work suggests that removing tar information on cigarette packs would have an impact on consumers’ perceptions of the relative harm of cigarettes in South Korea^11^.

The misperceptions of misleading claims and ‘low tar’ cigarettes have been known for some time. The FCTC, an evidence based public health treaty already recommends the prohibition of the reporting of quantitative measures of cigarette constituents due to its ability to mislead consumers. These findings suggest the need for stronger packaging regulations in South Korea to reduce the ability of tobacco companies to provide misleading information about their products.

In addition to deliberate marketing of tar on the cigarettes, these data indicate that tobacco manufacturers are employing misleading terms to further communicate the relative harms of their products. The marketing strategy shown here also includes the subtle use of color and brand variant names.

While what is considered a low tar cigarette is relative and based on the market, our data show that the market in South Korea, at least in Seoul and Busan, skews toward 1 mg. More brand variants were purchased with 1 mg machine-assessed tar yields than any other tar level. These ‘low’ and ‘ultra-low’ brand variants dominate the current cigarette landscape in Seoul and Busan. While specific details on the relative market share for all ultra-low and low tar cigarettes in South Korea are not available, analysis by Euromonitor International suggests that ‘lower risk’ cigarette products, such as those 1 mg or lower are a growing segment of the market, with 1 mg tar products dominating the best-selling cigarettes lists in South Korea^13^. Euromonitor International also reports that KT&G’s new 1 mg or lower cigarette products now account for one-third of the company’s sales, which is a considerable share of the current market^13^.

To our knowledge, this is the first study to systematically purchase and review the machine-assessed tar yields of South Korean cigarettes. These findings suggest that tobacco companies are emphasizing ‘ultra-low’ and ‘low’ tar variants by introducing many different variants of cigarettes in these categories and by reinforcing the machine-assessed tar yields with additional branding to communicate these lower tar levels.

### Limitations

There are some limitations of this study. While we did not purchase packs in low-income neighborhoods, data collection maximized the opportunities to purchase unique cigarettes by diversifying vendor types across all neighborhoods visited. The data collection methodology is designed to maximize the number of unique cigarette packs purchased, but may not fully represent all cigarettes offered for sale in South Korea. However, based on a comparison of packs purchased and Euromonitor data, we were able to confirm that we purchased the 15 top brand variants and 88% of the top 50 brands variants sold in South Korea, suggesting we captured a majority of the current cigarette market at the time of data collection. Thus, despite our data collection limitations, the large number of unique cigarette packs purchased means that most of the cigarette brand variants available for sale in South Korea were included in our analysis.

## CONCLUSIONS

As a signatory to the FCTC, and having ratified the treaty in 2005, it is important that South Korea work to further protect the public’s health by enacting more of the recommendations stated in Article 11. The FCTC recommends prohibiting the reporting of quantitative measures of cigarette constituents due to its ability to mislead consumers. This suggests the need for recommending stronger packaging laws not only in South Korea, but in all countries to reduce the ability of tobacco companies to provide misleading information about their products. Under Presidential Decree, the list of terms banned on cigarette packaging should be updated. The present misleading descriptors ban should be more explicit and include a requirement to not only remove the display of machine-assessed tar yields but also the types of additional branding that corresponds to and highlights those tar yields, as well as any misleading adjacent terms (smooth, mellow) that might suggest to consumers that one type of cigarette is less harmful than another.
