# Supplementary material for: Machine-assessed tar yield marketing on cigarette packages from two cities in South Korea
Source: Tob Induc Dis. 2021 Jun 25;19:54. doi: 10.18332/tid/136421 (PMC8231860; doi:10.18332/tid/136421)
Supplement: Supplementary file 1 [file TID-19-54-s1.pdf]

Table S1: Number of unique packs collected, by brand family and machine-assessed tar yield

| | Brand Family Name | Machine-assessed Tar Yield | | | | | | | | | | | | | | | |
|---|---|---|---|---|---|---|---|---|---|---|---|---|---|---|---|---|---|
| | | 0.1 | 0.5 | 1 | 1.5 | 2 | 3 | 3.5 | 4 | 4.5 | 5 | 5.5 | 6 | 6.5 | 7 | 8 | Total |
| KT&G | Esse | 1 | 2 | 17 | | | 1 | 1 | 1 | 1 | | 1 | | 1 | | | 26 |
| | Bohem | | | 8 | | | 3 | | | | 3 | | 5 | | | | 19 |
| | Raison | | | 4 | | | 6 | | | | | | 2 | | | | 12 |
| | The One | 2 | 1 | 5 | | | | | | | | | | | | | 8 |
| | Africa | | | | | | | | | | 5 | | | | | | 5 |
| | Simple | | | 1 | | | 1 | | | | 1 | | 1 | | | | 4 |
| | Time | | | | | | | 1 | | | | 2 | | | | | 3 |
| | This | | | | | | | | | | | 1 | | 1 | | | 2 |
| | Davidoff | | | | | | 1 | | | | | | 1 | | | | 2 |
| | Cloud 9 | | | 1 | | | | | | | 1 | | | | | | 2 |
| | Entz | | | | | | | 1 | | | | | | | | | 1 |
| | Seasons | | | | | 1 | | | | | | | | | | | 1 |
| | Tonino | | | | | | | | | | | | 1 | | | | 1 |
| | Hallasan | | | | | | | | | 1 | | | | | | | 1 |
| | Lilac | | | | | | | | | | | | | 1 | | | 1 |
| | **Total** | **3** | **3** | **36** | **0** | **1** | **12** | **3** | **1** | **2** | **10** | **4** | **10** | **3** | **0** | **0** | **88** |
| PM, Korea | Marlboro | | | 3 | | | 2 | | | | 2 | | 8 | | | 1 | 16 |
| | Parliament | | | 6 | 1 | | 1 | | 1 | | 3 | | | | | | 12 |
| | Virginia Slims | | | 2 | | | 1 | | | | 2 | | | | | | 5 |
| | Lark | | | 1 | | | | | | | | | | | | | 1 |
| | **Total** | **0** | **0** | **12** | **1** | **0** | **4** | **0** | **1** | **0** | **7** | **0** | **8** | **0** | **0** | **1** | **34** |
| BAT, Korea | Dunhill | 2 | | 10 | 4 | | 5 | | 3 | | | | 3 | | | | 27 |
| | Rothmans | | 1 | 2 | 1 | | | | | | | | 1 | | | | 5 |
| | **Total** | **2** | **1** | **12** | **5** | **0** | **5** | **0** | **3** | **0** | **0** | **0** | **4** | **0** | **0** | **0** | **32** |
| JTI, Korea | Mevius | | | 6 | | | 5 | | | | 1 | | 5 | | | 1 | 18 |
| | Camel | | | 1 | | | 1 | | | | 1 | | 1 | | | 1 | 5 |
| | Sevenstars | | | | | | | | | | | | | | 1 | | 1 |
| | **Total** | **0** | **0** | **7** | **0** | **0** | **6** | **0** | **0** | **0** | **2** | **0** | **6** | **0** | **1** | **2** | **24** |
| **Total** | | **5** | **4** | **67** | **6** | **1** | **27** | **3** | **5** | **2** | **19** | **4** | **28** | **3** | **1** | **3** | **178** |

Table S2: Number of unique packs collected with additional tar number branding, by brand family and machine-assessed tar yield

| | | Additional Tar Number Branding | | | | | | | | | |
|---|---|---|---|---|---|---|---|---|---|---|---|
| | Brand Family Name | 0.1 | 0.5 | 1 | 3 | 4 | 5 | 6 | 7 | 8 | Total |
| KT&G | Esse | 1 | 2 | 13 | | 1 | | | | | 17 |
| | Bohem | | | 6 | 1 | | 1 | 3 | | | 11 |
| | The One | 2 | 1 | 5 | | | | | | | 8 |
| | Simple | | | 1 | 1 | | 1 | | | | 3 |
| | Cloud 9 | | | 1 | | | 1 | | | | 2 |
| | Raison | | | 2 | | | | | | | 2 |
| | Tonino | | | | | | | 1 | | | 1 |
| | **Total** | **3** | **3** | **28** | **2** | **1** | **3** | **4** | **0** | **0** | **44** |
| JTI, Korea | Mevius | | | 6 | 5 | | 1 | 5 | | 1 | 18 |
| | Camel | | | 1 | 1 | | 1 | | | 1 | 4 |
| | Sevenstars | | | | | | | | 1 | | 1 |
| | **Total** | | | **7** | **6** | | **2** | **5** | **1** | **2** | **23** |
| BAT, Korea | Dunhill | 2 | | 6 | 5 | | | 3 | | | 16 |
| | Rothmans | | 1 | 2 | | | | | | | 3 |
| | **Total** | **2** | **1** | **11** | **5** | **0** | **0** | **3** | **0** | **0** | **22** |
| PM, Korea | Parliament | | | 5 | 1 | 1 | 3 | | | | 10 |
| | Virginia Slims | | | 2 | 1 | | 2 | | | | 5 |
| | Marlboro | | | 3 | | | 1 | | | | 4 |
| | Lark | | | 1 | | | | | | | 1 |
| | **Total** | **0** | **0** | **11** | **2** | **1** | **6** | **0** | **0** | **0** | **20** |
| **Total** | | **5** | **4** | **57** | **15** | **2** | **11** | **12** | **1** | **2** | **109** |

© 2021 Iacobelli M. et al.
